# Union of the European Phoniatricians’ position statement on the exit strategy of phoniatric and laryngological services: staying safe and getting back to normal after the peak of coronavirus disease 2019 (issued on 25th May 2020)

**DOI:** 10.1017/S002221512000122X

**Published:** 2020-06-16

**Authors:** A Geneid, T Nawka, A Schindler, H Oguz, V Chrobok, O Calcinoni, A am Zehnhoff-Dinnesen, K Neumann, M Farahat, T Abou-Elsaad, M Moerman, E Chavez, J Fishman, R Yazaki, B Arnold, Z Frajkova, S Graf, C Pflug, J Drsata, G Desuter, C Samuelsson, M Tedla, D Costello, E Sjögren, M Hess, T Kinnari, J Rubin

**Affiliations:** 1 Department of Otorhinolaryngology and Phoniatrics – Head and Neck Surgery, University of Helsinki and Helsinki University Hospital, Finland; 2 Department of Audiology and Phoniatrics, Charité – Universitätmedizin Berlin, Germany; 3 ‘L Sacco’ Department of Biomedical and Clinical Sciences, University of Milan, Italy; 4 Private practice, Ankara, Turkey; 5 Department of Otorhinolaryngology and Head and Neck Surgery, University Hospital Hradec Kralove, Charles University, Faculty of Medicine in Hradec Kralove, Czech Republic; 6 Voice and Music Professionals’ Care Team, Milan, Italy; 7 Clinic of Phoniatrics and Pedaudiology, University Hospital Münster, Westphalian Wilhelm University, Germany; 8 Department of Otolaryngology, Research Chair of Voice, Swallowing, and Communication Disorders, Head and Neck Surgery, College of Medicine, King Saud University, Riyadh, Saudi Arabia; 9 Phoniatric Unit, ORL Department, Faculty of Medicine, Mansoura University, Egypt; 10 Private practice, Sint-Martens-Latem, Belgium; 11 Centro de Foniatría y Audiología, Mexico City, Mexico; 12 Royal National Throat, Nose and Ear Hospital, University College London Hospitals NHS Foundation Trust, UK; 13 Artistic Voice Institute, Oswaldo Cruz German Hospital, São Paulo, Brazil; 14 Private practice, Munich, Germany; 15 Department of Otorhinolaryngology, Head and Neck Surgery, University Hospital and Comenius University Bratislava, Slovakia; 16 Otorhinolaryngology/ Phoniatrics, Klinikum rechts der Isar, Technical University Munich, Germany; 17 Department of Voice, Speech and Hearing Disorders, Center for Clinical Neurosciences, University Medical Center Hamburg-Eppendorf, Germany; 18 Voice and Swallowing Clinic, ENT Head and Neck Surgery Department, Cliniques Universitaires Saint-Luc, Louvain, Brussels, Belgium; 19 Department of Biomedical and Clinical Sciences, Division of Sensory Organs and Communication, Linköping University, Sweden; 20 Department of ENT, Wexham Park Hospital, Slough, UK; 21 Department of Otorhinolaryngology Head and Neck Surgery, Leiden University Medical Center, The Netherlands; 22 Deutsche Stimmklinik, Hamburg, Germany; 23 Royal National ENT and Eastman Dental Hospitals Division, University College London Hospital NHS Trust, UK

**Keywords:** Phoniatrics, Laryngology, Otorhinolaryngology, Voice, Swallowing, Pediatrics, Audiology, COVID-19

## Abstract

**Background:**

The following position statement from the Union of the European Phoniatricians, updated on 25th May 2020 (superseding the previous statement issued on 21st April 2020), contains a series of recommendations for phoniatricians and ENT surgeons who provide and/or run voice, swallowing, speech and language, or paediatric audiology services.

**Objectives:**

This material specifically aims to inform clinical practices in countries where clinics and operating theatres are reopening for elective work. It endeavours to present a current European view in relation to common procedures, many of which fall under the aegis of aerosol generating procedures.

**Conclusion:**

As evidence continues to build, some of the recommended practices will undoubtedly evolve, but it is hoped that the updated position statement will offer clinicians precepts on safe clinical practice.

## Introduction

The coronavirus disease 2019 (Covid-19) pandemic continues. However, new-onset infection and mortality rates appear to have come to a plateau or to have decreased in many countries. The following material is presented to inform the practices of ENT surgeons and phoniatricians in particular, in countries where clinics and operating theatres are reopening for elective work.

The following position statement from the Union of the European Phoniatricians, updated on 25th May 2020 (superseding the previous statement issued on 21st April 2020), contains a series of recommendations for phoniatricians and ENT surgeons who provide and/or run voice, swallowing, speech and language, or paediatric audiology services.

The opinions and recommendations below are the collective recommendations of the Union of the European Phoniatricians presented through the authors in their personal capacities. They are not necessarily the same in each of the authors' own hospital or clinic, neither do they necessarily represent the opinion of the authors’ hospital or practice. In addition, this position statement does not act as a policy that is enforceable, but rather as a collection of recommendations.

Healthcare providers are at high risk of contracting the severe acute respiratory syndrome coronavirus-2 (SARS-CoV-2) virus. Of the first 138 SARS-CoV-2 in-patients, 40 were healthcare workers.^1^ Among healthcare practitioners, ENT surgeons and phoniatricians are at increased risk.^2^ As a poignant example, the first documented death of a physician was that of an ENT surgeon in Wuhan, China, on 25th January 2020.^3^

This increased risk is due in part to exposure to aerosol generation,^4^ a result of the presence of the virus in the nasal and pharyngeal cavities of infected individuals. Viral load has been noted to be higher in the nose than the throat. This places practitioners who carry out nasendoscopic examinations at higher risk, including phoniatricians and ENT surgeons, particularly laryngologists.^5^

The risk of infection likely results not only from an infectious aerosol being directly inhaled, but also from it forming on contaminated surfaces, both during and after procedures. An increased risk of exposure has been found during head and neck examinations and endoscopies, and during interventions performed in the upper airway and food passages.^5–8^

Some asymptomatic individuals have been found to harbour active viral infection and to be potentially infectious. Viral load in infected but asymptomatic individuals has been noted to be similar to that in symptomatic patients.^5^ This is underscored by a study from a large German outbreak that showed 22.2 per cent of all infected individuals to be asymptomatic. Even in areas where there had been extensive testing, there may be five times as many unrecognised cases.^9^

Accordingly, personal protective equipment (PPE), including respiratory filtering facepiece code 3 (FFP3) masks, face and eye protection, caps, gloves and fluid-resistant gowns, should be used, even if the patient is totally asymptomatic, whenever the procedure includes examination or manipulation of the patient's throat, nose, larynx or upper airway. Video examples of donning PPE can be found online.^10,11^ The use of such equipment is important wherever such examinations take place, be it in community polyclinics, private clinics, hospital out-patient settings or wards, emergency departments, intensive care settings, or operating theatres.

As mentioned above, phoniatric and ENT clinics are now being reopened in many countries, with the proviso being that patient-related pathways and clinic or hospital-related guidelines must be followed. This applies generally, but is particularly essential in clinical scenarios that include potential aerosol generating procedures.

An aerosol generating procedure is a procedure that potentially stimulates the explosive expulsion of air via coughing, sneezing and so on that results in the release of airborne particles, thereby increasing the risk of airborne transmissions of infections that are classically spread by droplet transmission. Such procedures include, for example, but are not limited to: (1) tracheostomy operations, including percutaneous dilatation tracheostomy; (2) intubation and extubation; (3) open suctioning, positive airway pressure, jet ventilation, high-frequency ventilation and high-flow nasal oxygen; (4) bronchoscopy; (5) flexible or rigid endoscopic oral, transnasal, and laryngeal examinations, including office operations; (6) drainage of quinsy infections; (7) management of epistaxis; (8) removal of fish bones or other foreign bodies in the pharynx or nasal cavity; (9) transoral or transnasal injections into the larynx; (10) transoral or transnasal oesophagoscopy or gastroscopy; (11) face-to-face transcricothyroid or transoral injection into the larynx, pharynx or mouth; (12) face-to-face ultrasound-guided injections or procedures in the head and neck; and (13) face-to-face dental or oral cavity procedures.

The precautions and suggestions are discussed below under different clinical scenarios. ‘Telehealth’ in the text refers to instances in which both the care provider and the patient are communicating remotely through video and/or audio applications.

Based on the fact that the current situation may continue for months, if not longer, we recommend the following practices (as the new ‘norm’).

## General recommendations

Routine face-to-face examinations, office procedures and operations of an elective nature should only be undertaken on asymptomatic patients. Asymptomatic patients undergoing elective office procedures or operations under local or general anaesthesia that include mucosal surface puncture or injections should be SARS-CoV-2 tested 2–3 days before the operation, followed by quarantine until the operation day. Symptomatic patients should be regarded as Covid-19 positive until proven otherwise.

Video-endoscopy is preferred to ‘naked eye’ nasendoscopy. Topical anaesthesia of the nasal cavity is preferably carried out with anaesthetic-soaked cotton or gel rather than spray, or is avoided completely. After each procedure with patients gagging or coughing, there should be a minimal time interval of 30 minutes to allow for thorough cleaning of the facility. Such an interval may alter depending on the facilities in which the procedure takes place, most importantly the frequency of room air changes. This applies to nasendoscopy and video-laryngoscopy, office procedures, and fibre-optic endoscopic evaluations of swallowing. A paradigm shift is required, away from face-to-face examination and towards the provision of diagnostics and therapy through solutions embracing telehealth.

## Voice or airway practice

### Office examination

Routine office examinations can be recommenced for elective ENT procedures, and phoniatric patients and services, providing that the local Covid-19 pandemic-related situation has stabilised and guidelines are followed. Priority should be given to patients in whom there is the possibility of malignancy or airway compromise. Full PPE, including FFP3 masks, should be used, even during the examination of asymptomatic patients. This includes tracheostomy tube changes, and changes to or adjustments of voice prostheses.

### Office procedures

Office procedures under local (or no) anaesthesia that include puncture of the vocal folds or mucosal cover can be undertaken; however, full PPE including FFP3 masks must be worn, and the patient should be SARS-CoV-2 tested 2–3 days prior to the operation. Such operations include, for example, vocal fold augmentation and other procedures conducted through a channelled fibrescope. Ideally, such operations should be performed in ventilated, low-pressurised rooms.

### Laryngeal procedures under general anaesthesia

Laryngeal procedures in the operating theatre can be undertaken for asymptomatic patients, but they should be preceded by SARS-CoV-2 testing 2–3 days prior to the operation. Priority must be given to cases where a malignancy is suspected and the need for biopsies or curative surgery is imperative, or where the airway itself is at risk. Countrywide or local guidelines should be followed as to the prioritising of patients.

Operations such as tracheostomy, and those involving jet ventilation, high-flow humidified oxygen, laser, microdebrider and suction cautery, pose a greater risk to the surgeon and operating theatre personnel. Flow-controlled ventilation utilising small-bore, cuffed intubation tubes may offer a safer alternative to jet ventilation in certain circumstances. In general, these operations should be preceded by adaptation of the operating theatre to lower pressure. Over-pressure could increase the risk of viral spreading (in the instance of a patient with a false negative test result). Surgical suction devices and smoke evacuators should be equipped with filtration systems with greater than 99 per cent efficiency at removing particles sized 0.1 nm, thereby diminishing the risk of contamination during the operation. That said, filtration devices do not change the classification of an aerosol generating procedure.

### Patient testing

In an elective case scenario, and in the absence of symptoms that can be attributed to SARS-CoV-2, one negative test should be adequate. In an emergency scenario, testing or awaiting the result of a test should not delay an operation; however, SARS-CoV-2 positivity must be assumed.

In elective circumstances, patients with positive results, and in the absence of SARS-CoV-2 symptoms, should be retested to avoid delays due to false positive results. A confirmed positive result mandates the delay of surgery for at least two weeks and until a further test is negative (assuming such a delay does not entail danger to the patient's airway or life).

All emergency cases in which SARS-CoV-2 testing is not possible due to time constraints, or in which the test has come back as positive, should be considered to be SARS-CoV-2 positive. When operating on positive cases, PPE ideally includes powered air-purifying respirators for all personnel in the operating theatre.^12^

### Telehealth

Positive lessons of telehealth learned during the pandemic should continue to be utilised both in history taking and in provisions of voice and other phoniatric therapy to our patients. Face-to-face voice therapy should be changed to remote voice therapy. Smartphone applications should be utilised if available. Telehealth tools and real-time video communication must comply with national safety and security regulations.

### Botulinum injection patients

This section concerns patients receiving regular botulinum injections for voice spasms, tremors or vocal fold dysfunction. In asymptomatic patients, injections performed under electromyography (EMG) guidance that do not puncture the vocal fold mucosal covering can be undertaken, without prior SARS-CoV-2 testing, if all healthcare providers in the procedure room wear PPE including FFP3 masks. Such injections include, for example, those performed via a cricothyroid approach into the thyroarytenoid muscle. Similar recommendations apply to laryngeal EMG examinations. Patients should also wear surgical masks during the procedure. History taking and patient interview should be conducted utilising telehealth or in a room separate from the one in which the actual procedure is undertaken.

Injections carried out through a channelled fibrescope, and other injections likely to induce cough, for example those into the posterior cricoarytenoid muscle, or those through a transtracheal, translaryngeal or transoral route, should only be performed after a confirmed negative status on a SARS-CoV-2 test, but still under strict PPE adherence (because of the risk of a false negative result).

## Swallowing

Fibre-optic evaluations of swallowing and videofluoroscopic swallow studies are aerosol generating procedures. Such examinations require PPE including FFP3 masks.

In cases where there is a clear need for the assessment of swallowing, to enable decisions such as those relating to percutaneous endoscopic gastrostomy placement, it is preferable to use videofluoroscopy or modified fibre-optic endoscopic evaluations of swallowing with only one or two food and liquid consistencies, in order to minimise the duration of the actual procedure.

For new referrals, each department or team needs to determine the ‘time sensitivity’, ‘urgency’ and ‘preferred short protocol’ on a case-by-case basis. They must take into account each patient's medical condition, social circumstances and needs. Swallowing examination and therapy can potentially be started through remote telehealth options.

On the ward, Covid-19 positive patients with swallowing problems should be managed, if possible, through indirect assessment and treatment provided by the healthcare personnel who are caring for them. Remote telehealth and diagnostics should be used for such patients as much as possible.

In Covid-19 suspected patients, it is recommended that the healthcare provider stands 2 m away from the patient while evaluating oromotor function. Closer contact is required when assessing oral mucosa, palate and dentition. If closer contact is needed, the investigator should stand to the side of the bed, facing away from the patient. The PPE must be worn in accordance with local guidelines, but with a minimum of an FFP3 mask, visor, cap and fluid-resistant gown.

## Speech and language

Speech and language assessment should be undertaken partially or wholly through telehealth, whenever possible. Routine questionnaires that can be completed prior to the actual consultation should be used and be available to the examiner.

When treating children, the number of adults accompanying the child on each visit should be limited to one. When treating adults, they should be requested to visit the clinics alone, unless their underlying cognitive, motor or other medical conditions require carers to be present.

## Paediatric audiology

Children with acute conditions necessitating a visit to the hearing centre will need to be seen on a face-to-face basis. It is recommended that the ear examination and hearing assessment be undertaken as expeditiously as possible. Screening questionnaires should be completed by the parent prior to the consultation, in an attempt to assist the examiner and prioritise therapy.

Screening of newborn hearing should be maintained. This includes the Early Detection of Hearing Impairment assessment for neonates who are suspected (based on screening assessments) to have congenital hearing impairment.

In general, the duration of face-to-face clinical interactions between the clinician and child, and time spent waiting by the child in the clinic, should be kept to the minimum, with no waiting time at all if possible.

## Disinfection

Extensive attention should be given to disinfection of the examination rooms and the equipment within them. We recommend strict compliance with local health and safety regulations relating to sterilisation and disinfection. The use of disposable equipment is preferred wherever possible.

## Reimbursement

The Union of the European Phoniatricians recommends that telehealth and teleconsultations be recognised and reimbursed in accordance with local and national policies during this Covid-19 pandemic.

## Confidentiality

Confidentiality is paramount. General data protection regulations, local and regional variants, and appropriate legislation must be complied with. In particular, when undertaking a remote consultation, the examiner must: introduce themselves thoroughly, explain the rationale for the examination and obtain informed consent to proceed. They must document the examination in some form of a contemporaneous manner, and must send their findings (with permission of the patient or carer) to the referring general practitioner or physician and other appropriate individuals. The examiner must be aware of their duty of care, be alert to safeguarding issues and understand the mechanism of escalation.

- ENT and phoniatric practitioners undertake aerosol generating procedures, and are at high risk of contracting coronavirus disease 2019 (Covid-19)
- Asymptomatic carriers of the virus are commonplace, and false negative tests occur
- Thus, all surgical interventions must be viewed from the perspective of management of a Covid-19 positive patient
- Symptomatic patients should be regarded as Covid-19 positive until proven otherwise
- A paradigm shift is required, away from face-to-face examination, to provision of diagnostics and therapy through solutions embracing telehealth
- Asymptomatic patients should be tested 2–3 days before operations, followed by quarantine until operation day
